# The pig is an excellent model to determine amino acid digestibility of human foods and to generate data needed to meet human amino acid requirements

**DOI:** 10.3389/fnut.2024.1434430

**Published:** 2024-08-01

**Authors:** Hans H. Stein

**Affiliations:** Division of Nutritional Sciences, University of Illinois, Urbana, IL, United States

**Keywords:** additivity, amino acids, digestibility, pig, protein

## Abstract

The protein value of any food item is determined by the quantity and ileal digestibility of indispensable amino acids in that food. To determine the ileal digestibility of amino acids, an animal model needs to be used, and the pig is the preferred model because values for ileal digestibility obtained in pigs are representative of values obtained in humans. In addition, pigs are omnivorous animals like humans, they are meal eaters, they consume most diets that humans consume, they are easy to work with, and they can be used for repeated determinations of digestibility in many foods. It is, therefore, possible to use pigs to establish a database with digestibility values for human foods and by correcting digestibility values obtained in pigs for the basal endogenous losses of amino acids, it is possible to calculate true ileal digestibility values that are additive in mixed meals. As a consequence, the protein quality of a meal consisting of several food items can be calculated based on digestibility values obtained in pigs. Future work needs to focus on expanding existing databases for amino acid digestibility in foods to include more food items, which will make it possible to estimate the amino acid value of more mixed meals. It is also necessary that the amino acid values in mixed meals be related to requirements for digestible indispensable amino acids in the individuals consuming the meals. The current contribution describes the basic steps in determining amino acid digestibility in human foods using the pig as a model and also outlines future steps needed to further improve amino acid nutrition in humans.

## 1 Introduction

Animals have been used as models for humans in nutrition research for centuries and a number of important discoveries in nutrition were based on animal studies. The earliest recorded nutrient digestibility experiments were conducted approximately 270 years ago by de Reaumur who fed small, perforated metal tubes filled with grass to sheep [cited from Sauer et al. (1)]. Among animals, pigs are attractive models for humans because diet and intake patterns as well as diurnal patterns are similar to humans. The anatomy of the digestive system in pigs, secretions of enzymes and hormones, and absorption mechanisms in pigs are also very similar to the human digestive system (2, 3). Pigs are also omnivorous animals like humans, they are meal eaters, and they will eat pretty much anything humans eat, which makes it possible to study digestion, absorption, and post-absorptive metabolism of nutrients in pigs and apply results to humans (2). Indeed, in experiments where the same proteins were consumed by pigs and humans, it was demonstrated that for all indispensable amino acids, the true ileal digestibility is very similar (3, 4). Given that pigs are easy to work with, easily tolerate procedures to collect fluids from the distal ileum, and can be fed human diets without modifications, it is natural that the pig has emerged as the preferred model to study amino acid digestibility in humans (5, 6). The digestibility of amino acids is less in newly weaned pigs than in older pigs (7), whereas no differences between growing pigs and mature pigs have been observed (8), and a growing female pig between 30 and 100 kg has, therefore, been proposed as an appropriate model for humans (6). There are no indications that amino acid digestibility is different between male and female pigs, but because most male pigs in commercial units are castrated, female pigs are usually utilized in experiments to determine digestibility of amino acids in human foods. There are no indications that differences in ileal digestibility of amino acid among commercial breeds of pigs exist and the choice of breed is likely not going to influence digestibility. During the last decade, there has, therefore, been a number of experiments conducted in which pigs were used to determine digestibility of amino acids in human foods and results have been used to calculate the digestible indispensable amino acids score (DIAAS) in a large number of human foods. As a consequence, a large set of data with values for the ileal digestibility of amino acids in human foods determined in pigs is now available (9), and more data will undoubtedly be generated in the future. There is, therefore, a need to highlight some of the consequences of determining amino acid digestibility in human foods using pigs as models. The practical aspects of preparing, managing, and feeding pigs used in digestibility experiments have been highlighted in two recent publications (6, 10). Likewise, detailed procedures for calculation of true ileal digestibility of amino acids and values for DIAAS have also been provided (5, 11) and detailed descriptions of the factors used to calculate DIAAS have been provided (12). There is, however, a lack of information about the application of digestibility data for amino acids obtained in pigs into practical recommendations for human consumption. It is, therefore, the objective of the present contribution to provide examples of how the pig model can be used to not only generate digestibility values for amino acids, but also how these data can impact formulation of meals for humans to meet requirements for amino acids. It is not the objective to give an exhaustive review about factors affecting amino acid requirements in humans, nor is it the objective to discuss post-absorptive metabolism of amino acids. Instead, the focus will be on discussing why amino acid digestibility is important and how data for amino acid digestibility obtained in pigs may be used in human food formulation.

## 2 The importance of amino acids in nutrition

Although it is generally assumed that humans have requirements for protein, this is not entirely true, because humans, like other monogastic species, have requirements for indispensable amino acids and not for protein *per se* (13). Of the 20 amino acids that are needed for protein synthesis, the body can synthesize only 10 in quantities that are sufficient to meet the requirement, whereas the remaining amino acids need to be supplied in the diet; these amino acids are, therefore, called dietary indispensable. There is no storage in the body of excess amino acids, and the 10 indispensable amino acids, therefore, need to be provided in the diet each day. In fact, recent evidence indicates that providing approximately one third of the daily requirements for indispensable amino acids at each meal supports muscle protein synthesis to a greater extent than providing the majority of the amino acids in one daily meal (14, 15). It is therefore most important that sufficient quantities of the indispensable amino acids are provided in each meal every day. However, not all amino acids in food proteins are digested, but only the amino acids that are digested and absorbed contribute to the protein status of the individual. It is therefore the digestible quantity of each indispensable amino acid in each meal that determines if the requirement for protein synthesis can be met. Whereas there are estimates for requirements of total amino acids by different age groups (13) there is a lack of estimates for requirements for ileal digestible amino acids. However, most experiments conducted to determine amino acid requirements used diets that were high in animal proteins, and the true ileal digestibility of amino acids in animal protein is generally very high (16–19). As an example, in 23 beef and pork ingredients, the true ileal digestibility of all indispensable amino acids was between 92 and 99% (18, 20, 21), and the same was the case for the digestibility of amino acids in whole milk (19). Assuming that human requirements for total amino acids are based primarily on animal proteins, the requirement for true ileal digestible amino acids may be estimated to be around 95% of the requirement for total amino acids (Table 1). The challenge, therefore, is to mix dietary food items at each meal to meet requirements for digestible quantities of each amino acid. As a consequence, a database with values for the digestibility of each amino acid in each food item is required (9).

**Table 1:** Calculated requirement for true ileal digestible amino acids, mg/kg body weight per day.^a^

Age, years	0.5	1–2	3–10	11–14	15–18	>18
Histidine	21	14	11	11	10	10
Isoleucine	34	26	22	21	20	19
Leucine	69	51	42	42	40	37
Lysine	61	43	33	33	31	29
SAA^b^	29	21	17	16	15	14
AAA^b^	56	38	29	29	27	24
Threonine	32	22	17	17	16	14
Tryptophan	9.0	6.1	4.6	4.6	4.3	3.8
Valine	47	34	28	28	27	25

a Data were calculated from World Health Organization (13) assuming a true ileal digestibility of 95% of amino acids used to determine requirements for total amino acids.

b SAA, sulfur amino acids, i.e., methionine and cysteine; AAA, aromatic amino acids, i.e., phenylalanine and tyrosine.

## 3 Procedures for determining digestibility of amino acids in food items fed to pigs

Proteins cannot be absorbed but need to be digested by gastric, pancreatic, and intestinal proteases to liberate the individual amino acids, which can then be absorbed. However, absorption of amino acids takes place only in the small intestine and proteins that have not been digested prior to the distal part of the small intestine, which is called the ileum, make no contribution to amino acid absorption because amino acids are not absorbed from the hindgut (8). It is therefore necessary to gain access to the digesta leaving the small intestine at the end of the ileum and a number of techniques have been suggested for this purpose (22). However, installment of a T-cannula at the distal ileum, which was first suggested 50 years ago (23), has been recognized as the most practical way to gain access to ileal digesta. A cannula in stainless steel or titanium is usually used although cannulas in polyethylene have also been proposed (23, 24). However, the inflexibility of the stainless steel or titanium cannulas has proven to result in better stability of the cannulas and less problems with dislodgements (25). The cannula consists of a flange that is inserted in the small intestine, and a barrel that penetrates the body wall. The upper part of the barrel is threaded, and the cannula is secured on the outside with a washer that is screwed onto the barrel. A screw cap is used to close the barrel and is removed when digesta is collected from the barrel (25). Cannulas with different dimensions can be used for different sizes of pigs, but for pigs from 30 to 100 kg, a cannula with an inner diameter of 2.24 cm and a barrel length of 6 cm is usually used (Table 2). The cannula is installed using a simple surgery that can be performed in less than 30 min by a trained surgeon (24, 25). Following surgery, pigs are housed individually to prevent other pigs from disrupting the cannula. Pigs are placed in a pen that should provide at least 1.25 square meter of space, and it is recommended that floors are fully slatted to prevent accumulation of fecal materials in the pen. If pens are not fully slatted, frequent cleaning is necessary to prevent coprophagy because if pigs ingest even small amounts of feces, which have a high concentration of the indigestible marker, calculations of amino acid digestibility will be inaccurate. No bedding is provided (6) because that may interfere with endogenous amino acid secretions and calculations of amino acid digestibility.

**Table 2:** Dimensions of intestinal cannula installed in pigs from 30 to 100 kg and used to determine ileal digestibility of human foods.

Item	Cannula for 30–100 kg pig
Barrel length, cm	6.00
Barrel outer diameter, cm	2.54
Barrel inner diameter, cm	2.24
Flange length, cm	7.00
Flange width^a^, cm	2.54

a The width of the flange is 2.54 cm in the middle, but the flange is tapered toward the extremes where the width is only 1.50 cm.

Pigs are typically given 7 days to recover after the surgery and feeding of experimental diets can then be initiated. An adaptation period of 5 days to experimental diets is recommended with ileal digesta being collected for 9 h per day on days 6 and 7 (6, 10). This schedule fits a normal work week, and collections can be scheduled to take place in the middle of the week. However, because amino acid digestibility is rapidly adjusted to the diet being provided, 3 days of adaptation is sufficient to obtain steady state in terms of marker and amino acid flow (26). In cases where the amount of an ingredient is limited, a 3-day adaptation period can, therefore, be considered.

Collection of ileal digesta from the cannula will not result in total collection and it is, therefore, necessary to include an indigestible marker in the diets being fed and ileal digestibility is subsequently calculated using the marker to estimate the flow of amino acids to the distal ileum (11). The assumption for using this procedure is that the marker is completely mixed with the test diet and that the marker flows through the intestinal tract at the same speed as undigested material, and these assumptions have been confirmed in several experiments. In most circumstances, titanium dioxide is used as the marker to determine the ileal digestibility of amino acids in human foods and an inclusion rate of 0.50% (dry matter basis) is often used (10). Where diets are provided in a meal form or as a porridge, it is usually not a problem to ensure a complete mixture of the marker and the diet (16, 27). Likewise, if the digestibility of amino acids in baked products such as bread or bagels is determined, the marker can be mixed into the dough and consumed along with the diet and subsequently analyzed in the ileal digesta. The marker can also easily be mixed into liquid diets such as milk or juice. However, for food items such as meat products, nuts, vegetables, and others, a complete incorporation of the marker with the meal may not be possible. It is recommended to provide all meals to pigs in the same form as they are usually consumed by humans (6), but to ensure a complete mixture of the marker with the meal, a gentle grinding may sometimes be necessary (28) in which case the ingredients are not fed to the pigs exactly as they would be consumed by humans. However, because mixing of the marker with the diet is critical for correct calculation of digestibility values, this compromise may sometimes have to be made and because amino acid digestibility is not impacted by the particle size of the ingredient ingested (29, 30), it is unlikely that this modification will have any impact on results. Another approach that can be used to ensure that the marker is well mixed into the meal is to incorporate the marker into a protein free mixture that is usually added to the diets to provide vitamins and minerals. Sometimes, it is also necessary to add protein free ingredients such as starch, lactose, oil, or sugar to this mixture to provide sufficient calories to the animals along with the protein food that is used (6). In this case, the protein food, which can be a meat product, can be cut into small squares prior to feeding and then gently mixed with the protein free mixture that also contains the marker. Because pigs are fed restrictedly and usually consume their meals quickly after feeding, this approach results in satisfactory incorporation of the marker in the meal and digestibility values using this approach, therefore, are associated with low errors (20, 21, 31).

After collection of the ileal digesta, it is critical that microbes in the digesta are quickly inactivated to prevent fermentation of amino acids after collection. It has been suggested that microbial activity can be prevented by adding an acid to the collection bags (6), but results of recent research demonstrate that this is not necessary because the acid is not mixed with the digesta flowing into the bags. Instead, if collection bags are frequently changed (i.e., every 30 min) and if the collected digesta are stored at −20°C immediately after collection, there is no advantage of adding acids to collection bags (32). Having a freezer located in the barn where pigs are kept is, therefore, critical.

At the conclusion of the collection period, the frozen digesta need to be thawed, mixed, and subsampled, and a subsample of around 200 mL is lyophilized. It is important to lyophilize these samples rather than oven dry them, because oven drying results in loss of amino acids and subsequently inaccurate calculation of amino acid digestibility (33). The lyophilized sample is ground using a coffee grinder, mixed, and a subsample is collected for analysis of dry matter, crude protein, amino acids, and titanium. Following analysis, values for apparent ileal digestibility, true ileal digestibility and digestible indispensable amino acid scores are calculated (5, 11).

## 4 Additivity of values for amino acid digestibility

Both animals and humans usually consume diets that consist of more than one source of amino acids and to meet requirements for digestible amino acids, it is critical that the values for amino acid digestibility that are determined are additive in mixed meals. However, values for the apparent ileal digestibility of amino acids are not additive in mixed diets (34), which prevents the use of such values in calculating the intake of digestible amino acids from a given meal. The lack of additivity of values for apparent ileal digestibility is caused by the influence of the endogenous amino acids on the ileal output of amino acids. The presence of endogenous nitrogen, or metabolic fecal nitrogen, in the feces of rats fed protein free diets was demonstrated in some of the earliest experiments to determine amino acid digestibility (35, 36). It was later demonstrated that the amount of endogenous nitrogen in the feces as a percentage of total fecal nitrogen output of rats fed a protein-containing ingredient depended on the inclusion rate of that ingredient in the meal (37), and subsequent work confirmed that values for the apparent ileal digestibility of amino acids are also influenced by the inclusion level of the ingredient in the diet (38, 39). As a consequence, it is necessary to correct values for the apparent ileal digestibility of amino acids for the pre-cecal endogenous loss of amino acids and subsequently calculate values that are independent of the inclusion rate of each ingredient in the diet. Values for endogenous losses of amino acids that are needed for this correction are obtained after feeding a protein free diet, and factors influencing ileal endogenous amino acid loses have been reviewed (40). Correcting values for apparent ileal digestibility for endogenous losses results in calculation of values for standardized ileal digestibility values, which is the term mostly used in animal feeding (11) whereas in human nutrition, values calculated after correction for ileal endogenous losses are termed true ileal digestibility values (5). However, strictly speaking, correction for values obtained after feed a protein free diet does not result in calculation of values for the true ileal digestibility of amino acids (11). Additivity of values for the standardized ileal digestibility of amino acids in mixed diets fed to pigs has been demonstrated multiple times (34, 41). Likewise, additivity of values for true ileal digestibility of food proteins in a mixed meal has also been demonstrated (19, 31, 42) and it is, therefore, possible to calculate the digestibility of indispensable amino acids in mixed meals from digestibility values for each amino acid in individual ingredients (43, 44). As a consequence, establishment of a database with values for the true (or standardized) ileal digestibility of amino acids for individual food items will allow dietitians and food professionals to calculate the quantities of digestible amino acids that are present in mixed meals and by comparing these quantities to requirements for amino acids, it can be determined if the meal is adequate in all indispensable amino acids. Establishing a food database with digestibility values for as many food proteins as possible, therefore, is critical (9, 45), and the only practical way to generate such a database is to use pigs to determine values for digestibility. As an example, ileal digestibility values for some food items determined in the authors laboratory using the procedures outlined above are presented in Tables 3, 4. However, there is a need to extend this database to contain a much larger number of food items.

**Table 3:** True ileal digestibility (%) of indispensable amino acids in selected plant food items determined in the authors laboratory.^a^

Food item	His	Ile	Leu	Lys	Met	Cys	Phe	Tyr	Thr	Trp	Val
**Grains**											
Maize	83	76	84	75	90	77	83	80	71	70	75
Barley, de-hulled	81	76	79	74	78	80	82	77	72	84	77
Oats, de-hulled	88	87	87	85	90	77	88	81	81	82	85
White rice, polished	91	92	94	92	95	94	95	92	91	95	94
Rye	77	72	74	67	81	76	79	66	94	75	71
Sorghum	74	74	76	69	77	68	76	71	68	73	74
Wheat	84	79	81	73	85	83	84	77	65	84	75
**Processed grain products**											
Corn flakes	88	93	97	78	98	93	95	95	93	91	94
Quick oats	88	87	88	83	89	90	88	88	85	85	86
Burger bun	92	91	93	64	93	92	94	90	88	95	90
**Grain protein concentrates, isolates**											
Oat protein concentrate	81	83	85	86	83	86	86	86	82	95	82
Brown rice protein concentrate	80	79	78	74	71	68	80	74	78	90	79
Pea protein concentrate	94	92	93	95	88	73	93	91	89	91	89
Rapeseed protein isolate	91	71	73	82	79	85	72	66	72	76	72
Rapeseed protein isolate, heated	98	95	97	95	98	94	97	96	94	98	95
Soy protein isolate	97	94	93	97	95	91	95	96	91	98	93
**Nuts**											
Pistachio nuts	89	87	88	87	87	88	87	88	88	92	88
Pistachio nuts, roasted	79	78	79	77	80	80	77	78	77	85	78
**Plant based meat analogs**											
Impossible burger patty	96	94	94	96	95	77	95	95	90	99	94
Beyond burger patty	90	90	90	94	84	64	92	92	86	98	89

a Data from the following references: Fanelli et al. (19), Cervantes-Pahm et al. (27), Bailey and Stein (28), Fanelli et al. (31), Abelilla et al. (46), Bailey et al. (47).

**Table 4:** True ileal digestibility (%) of indispensable amino acids in selected animal food items determined in the authors laboratory.^a^

Food item	His	Ile	Leu	Lys	Met	Cys	Phe	Tyr	Thr	Trp	Val
**Pork products**											
Pork belly, raw	99	98	98	99	98	93	98	98	98	99	97
Smoked bacon	97	97	97	97	97	89	96	97	96	97	95
Smoked cooked bacon	95	96	97	98	97	88	96	96	96	96	95
Ham, non-cured	94	94	95	96	96	77	94	95	93	92	93
Ham, cured	96	97	97	98	98	89	97	97	97	95	96
Pork loin, cooked to 63°C	96	96	97	97	97	85	96	97	96	95	95
Pork burger patty, 80% lean	98	96	97	98	97	79	96	95	95	100	96
**Beef products**											
Salami	95	96	96	96	96	91	95	96	96	96	96
Bologna	97	97	97	97	96	91	96	96	95	98	96
Beef jerky	96	97	97	96	97	90	97	97	96	97	96
Ground beef, raw	99	98	99	98	98	96	98	99	99	98	97
Ground beef, cooked	96	97	97	98	98	88	97	97	97	97	97
Rib eye roast, cooked to 63°C	96	96	96	96	97	88	95	96	94	95	95
Beef burger patty, 80% lean	93	94	94	96	96	63	93	92	89	95	92
**Milk products**											
Whey protein isolate	100	98	99	98	98	98	98	99	94	100	97
Whey protein concentrate	97	97	98	96	97	95	96	96	91	98	95
Milk protein concentrate	99	96	98	96	97	85	97	98	96	97	94
Dried skimmed milk	99	95	98	96	99	99	99	99	96	97	97
Skimmed milk powder	94	89	94	95	96	73	94	95	82	91	90

a Data from the following references: Mathai et al. (16), Fanelli et al. (19), Bailey et al. (20, 21), Fanelli et al. (31).

## 5 Application of amino acid digestibility values to human foods

The concept of determining protein quality in human foods is not new and was first attempted by establishing the protein efficiency ratio (PER) in foods (48). This procedure was based on determining the growth of rats fed different proteins and the PER value was calculated by expressing the growth over 28 days relative to the protein intake of the rats during those 28 days. A later modification to the procedure involved comparing all proteins to the PER of rats fed a casein-based diet and resulted in calculation of the casein-corrected PER. A different procedure called the biological value of proteins was based on the proportion of retained nitrogen relative to absorbed nitrogen and offered some advantages over the PER procedure (49). However, because of the very high requirement for the sulfur-containing amino acids by rats compared with humans, procedures using growth or nitrogen retention in rats have been criticized for not being reflective of the protein quality of foods for humans (50). As a consequence, protein evaluation based on the digestibility of nitrogen rather than retention or growth was introduced and this procedure also for the first time introduced values for the digestibility of individual amino acids (50). The procedure was called the “Protein Digestibility Corrected Amino Acid Score” (PDCAAS) and is used for regulatory purposes in the United States. The PDCAAS procedure also introduced the concept of scoring values of proteins by comparing quantities of digestible indispensable amino acids to the profile of amino acids required by children from 2 to 5 years (50), and therefore, recognized that humans have requirements for individual indispensable amino acids rather than for protein. The limitations of the PDCAAS procedure have been highlighted (16, 51) and resulted in recommendation of calculating DIAAS of proteins (5). The DIAAS procedure corrects some of the flaws in the PDCAAS procedure including measuring the ileal digestibility of each individual amino acid rather than the total tract digestibility of nitrogen. There are also several other advantages to the DIAAS procedure over the PDCAAS procedure, and one of the consequences of determining DIAAS values is that the pig is a more natural model for humans than the rat for reasons outlined above. In addition, because DIAAS is based on the ileal digestibility of each individual amino acid after correction for endogenous losses, the methodology for determining amino acid digestibility is identical to that used to determine digestibility of feed ingredients used in the feeding of animals. As a consequence, because values for true ileal digestibility are additive in mixed diets, DIAAS of meals consisting of several food items can be calculated, which is a great advantage because more than one protein item is included in most meals. However, DIAAS values, like PDCAAS values and PER values, only indicate the quality of a specific protein or meal, but do not indicate anything about the quantity needed to meet amino acid requirements. There is, therefore, a need for a further refinement of the DIAAS concept to directly link values to quantities of digestible amino acids required by different groups of humans. As a consequence, future work needs to focus on not only measuring digestibility of individual amino acids and calculating DIAAS values, but also on developing methodologies that can calculate the quantities of specific meals needed to meet amino acid requirements for humans.

## 6 Conclusion

Protein evaluation of human foods needs to start with determining the true ileal digestibility of each individual indispensable amino acid. The pig has proven to be an accurate model for humans in terms of amino acid digestibility and because pigs are easy to work with and easily tolerate the procedure of installing and maintaining an intestinal cannula in the distal ileum, it is easy to conclude that the pig is the preferred model for humans when it comes to amino acid digestibility determinations. Pigs easily consume most human foods in the form they are consumed by humans and can be used for multiple measurements of digestibility of amino acids. Detailed procedures for determining ileal digestibility of amino acids in pigs are available and the ileal digestibility of a number of food items determined in pigs have been published and can be used to determine DIAAS values in mixed meals. Future work will focus on development of methodologies that can connect DIAAS in individual ingredients and meals to the requirements for digestible indispensable amino acids in different groups of humans.

## Author contributions

HHS: Conceptualization, Methodology, Project administration, Supervision, Writing – original draft, Writing – review & editing.
